# Staged correction of pulmonary atresia, ventricular septal defect, and collateral arteries

**DOI:** 10.1111/jocs.16299

**Published:** 2022-02-09

**Authors:** Pieter van de Woestijne, Mostafa Mokhles, Ingrid van Beynum, Peter de Jong, Jeroen Wilschut, Ad Bogers

**Affiliations:** ^1^ Department of Cardio‐Thoracic Surgery Erasmus University Medical Center Rotterdam The Netherlands; ^2^ Department of Pediatric Cardiology Erasmus University Medical Center Rotterdam The Netherlands; ^3^ Department of Adult Congenital Cardiology Erasmus University Medical Center Rotterdam The Netherlands

**Keywords:** outcomes, pulmonary atresia, ventricular septal defect

## Abstract

**Objectives:**

Pulmonary atresia (PA) with ventricular septal defect (VSD) and systemic‐pulmonary collateral arteries (SPCAs) presents with variable anatomy with regard to the pulmonary vasculature, requiring personalized surgical treatment. A protocol consisting of staged unifocalization and correction was employed.

**Methods:**

Since 1989, 39 consecutive patients were included (median age at first operation 13 months). In selected cases, a central aorto‐pulmonary shunt was performed as the first procedure. Unifocalization procedures were performed through a lateral thoracotomy. Correction consisted of shunt takedown, VSD closure, and interposition of an allograft between the right ventricle and the reconstructed pulmonary artery. Echocardiographic data were obtained postoperatively and at interval follow‐up.

**Results:**

In 39 patients 66 unifocalization procedures were performed. Early mortality was 5%. Seven patients were considered not suitable for correction, of which four have since died. One patient is awaiting further correction. A correction was performed successfully in 28 patients. Operative mortality was 3% and late mortality was 11%. Median follow‐up after the correction was 19 years. Eleven patients required homograft replacement. Freedom from conduit replacement was 88%, 73%, and 60% at 5, 10, and 15 years respectively. Right ventricular function was reasonable or good in 75% of patients. All but one patient were in NYHA Class I or II.

**Conclusions:**

After complete unifocalization 30/37 patients (81%) were considered correctable. The staged approach of PA, VSD, and SPCAs results in adequate correction and good functional capacity. RV function after correction remains reasonable or good in the majority of patients.

## 1 INTRODUCTION

During the last 30 years, the surgical strategy for pulmonary atresia (PA) with ventricular septal defect (VSD) and systemic‐pulmonary collateral arteries (SPCAs) has been the subject of ongoing debate. There are several reports using the multi‐staged approach as well as reports on two‐staged approach or one‐stage midline primary repair.^1^, ^2^, ^3^, ^4^, ^5^, ^6^ Furthermore, it has been reported that unifocalization may not provide any long‐term benefit in terms of late survival.^7^

Historically patients with PA, VSD, and SPCAs were treated in our hospital with various surgical interventions depending on their clinical condition and previous cardio‐surgical procedures on presentation to our clinic. Since 1989, a protocol as described previously was followed in all our patients presenting with PA, VSD, and SPCAs.^8^ This protocol consisted of staged unifocalization procedures with a subsequent total correction, with the closure of the VSD and placing a pulmonary homograft between the right ventricular outflow tract (RVOT) and the pulmonary bifurcation. We report our results of a 30‐year experience with this staged protocol applied to all consecutive patients.

## 2 MATERIAL AND METHODS

Since 1989, thirty‐nine consecutive patients (21 boys and 18 girls) were included in this protocol. A 22Q11 deletion was diagnosed in 10 patients. The median age at the time of the first unifocalization was 13 months (range 2 weeks–189 months). Diagnostic catheterization was performed to assess the pulmonary vascularity and perfusion. In 13 patients with a hypoplastic central confluent pulmonary artery a central aorto‐pulmonary shunt was performed, intended to allow the confluent pulmonary artery to grow to improve the starting point for unifocalization.

Unifocalization was performed through a lateral thoracotomy with identification of all collateral arteries at that side. When adequate intrapulmonary connection was confirmed on preoperative angiography or intra‐operatively, the dual supply SPCA could be closed. When such connection was not established, the collateral artery was anastomosed to the native pulmonary artery as close as possible to the hilar pulmonary vasculature. In the case of an absent confluent pulmonary artery and in cases where further augmentation was indicated, a modified Blalock‐Taussig shunt was constructed to the ipsilateral subclavian artery. In 27 patients, an additional unifocalization procedure on the contra‐lateral side was performed to augment the blood supply to that lung. Patients underwent angiography before and after each procedure.

To evaluate the growth of the pulmonary arterial system, we retrospectively measured the pre‐ and postunifocalization Nakata‐index.^9^

The change in lung perfusion on angiogram pre‐ and postunifocalization was studied retrospectively to evaluate the outcome of the procedure. We studied the total lung perfusion including perfusion by the SPCAs versus the lung perfusion by flow through the confluent pulmonary artery alone.

Patients were selected for total correction based on the angiography findings and measurement data from catheterization. Pulmonary hypertension or unfavorable anatomical result of unifocalization at angiogram were contraindications for total correction. The total correction was performed through a median sternotomy with the use of extracorporeal circulation and moderate hypothermia. The modified Blalock‐Taussig and central shunts were divided. The VSD was closed with a Gore‐Tex® patch and a cryopreserved pulmonary homograft was interposed between the RVOT and the proximal pulmonary arterial system. Postoperative recovery and hospital or 30‐day mortality are reported.

Long‐term follow‐up was obtained from the records. In 24 survivors with a complete repair, echocardiographic data were available with the exception of one patient who was lost to follow‐up. Magnetic Resonance Imaging (MRI) data for 17 patients after the successful correction was available to assess right ventricular function.

This study was approved by the Ethical Committee with no need for informed consent.

Statistical analysis was performed using Statistical Package for the Social Sciences (SPSS) software, version 24.0 (SPSS Inc.). Frequencies were given as absolute numbers and percentages. The data were expressed as median with range. The paired *t*‐test was performed for statistical analysis. We applied the *χ* ^2^ test to compare frequencies in the two groups. The Kaplan–Meier method was applied to estimate freedom from reintervention and for survival. A *p* value less than .05 was considered to indicate statistical significance.

## 3 RESULTS

### 3.1 Unifocalization

After initiation of the unifocalization program, older patients were retroactively introduced into the cohort, reflected by a higher age at first surgery and the age at correction. During the first 5 years, the mean age at first unifocalization was 5.9 years (range 3 months–14 years) and after this period the mean age was 1.8 years (range 1 month–16 years with only one older patient of 16 who came to our hospital from abroad). Thirty‐nine consecutive patients with PA, VSD, and SPCAs were entered in the protocol. Of these 39 patients, 13 patients received a central shunt as the first procedure followed by 66 unifocalization procedures in which 129 collateral arteries were treated either with unifocalization or ligation. In seven patients a collateral artery was closed percutaneously.

In 50 unifocalization procedures, a modified Blalock–Taussig shunt was placed employing a 5–10 mm diameter tube, depending on the size of the patient. Later procedures most commonly featured a 5 mm tube prosthesis.

Based on several reports and our own clinical experience of the behavior of SPCAs, we tend to introduce more intrapulmonary anastomoses to avoid long segments of remaining SPCA tissue which could lead to stenosis or dilatation.^10^, ^11^

In one patient, the modified Blalock–Taussig shunt became obstructed 2 weeks after unifocalization, and the patient subsequently died after a reoperation in which the modified Blalock–Taussig shunt was replaced. One other patient died in hospital 3 months after unifocalization from an unknown cause.

The median time between the first and second unifocalization was 8 months (range 2 weeks–48 months), where the second unifocalization was considered indicated in 27 patients. A second surgical unifocalization was not necessary for twelve patients. Four patients had SPCAs primarily on one side. Five patients received a coil closing an SPCA on the contralateral side after unifocalization on the other side. After the first unilateral unifocalization, one patient still had incomplete vascularization of both lungs and progressive pulmonary hypertension, and was consequently treated with a central aorto‐pulmonary shunt on the contralateral side. One patient died before the second unifocalization, likely due to pulmonary infection with a respiratory syncytial virus 3 months after unifocalization.

After unifocalization 20 additional procedures were required. Apart from the coil closure of SPCAs, several other interventions were performed. In four patients a perigraft seroma around the modified Blalock–Taussig shunt was removed.^12^ In one patient the modified Blalock–Taussig shunt was incorrectly placed on the pulmonary vein, and was revised 2 weeks later. Two patients received a modified Blalock–Taussig shunt and a further two received a central shunt, owing to the presence of cyanosis after the second unifocalization. In one patient a transannular patch was placed between the RVOT and the pulmonary trunk, which was later followed by balloon dilatation and stent implantation in the left pulmonary artery. In two patients balloon dilatation of the modified Blalock–Taussig shunt was performed, with one patient also undergoing stent implantation. Two patients underwent revision of a central shunt with augmentation of the pulmonary artery, one with stent implantation and the other with balloon dilatation of the left pulmonary artery. One patient underwent revision of the modified Blalock‐Taussig shunt because of shunt occlusion. One patient underwent a sliding plasty of the distal trachea for stenosis with complete tracheal rings.

### 3.2 Nakata‐index and lung perfusion

The median Nakata‐indices (*n* = first 22 consecutive patients), indicating the relative size of the pulmonary arteries were 143 (range 49–345) before and 190 (range 49–401) mm^2^/BSA after unifocalization (*p* = .097). The normal value of this PA‐index is 330 mm^2^/BSA.^9^

From 30 patients we obtained detailed information about their lung perfusion by studying their cardiac catheterization images before and after their unifocalization procedure(s). The median total lung perfusion was 99% (range 80%–100%) before and 97% (range 80%–100%) after unifocalization. Median lung perfusion through the confluent pulmonary artery was 52% (range 0%–100%) before and 89% (range 40%–100%) after unifocalization. Although there seems to be an increase in perfusion through the confluent pulmonary artery, this difference was not statistically significant (*p* = .12), likely due to small group sizes.

### 3.3 Corrective surgery

About 30 of the 37 patients (81%) were considered correctable. One patient is awaiting correction after complete unifocalization. Twenty‐nine patients underwent a total correction as described earlier which was successful in 28 patients (97%). Median age at correction was 2.6 years (range 1–17 years) and median interval between unifocalization and correction was 8 months (range 1–48 months). The VSD was closed in all patients. One patient required a takedown during operation because of suprasystemic pressures of the right ventricle making it impossible to wean the patient from the extracorporeal circulation. In retrospect we didn't find clear factors predicting this failure in comparison with other patients. One patient had a resternotomy for persistent bleeding the same day and recovered without complications.

**Figure 1:**
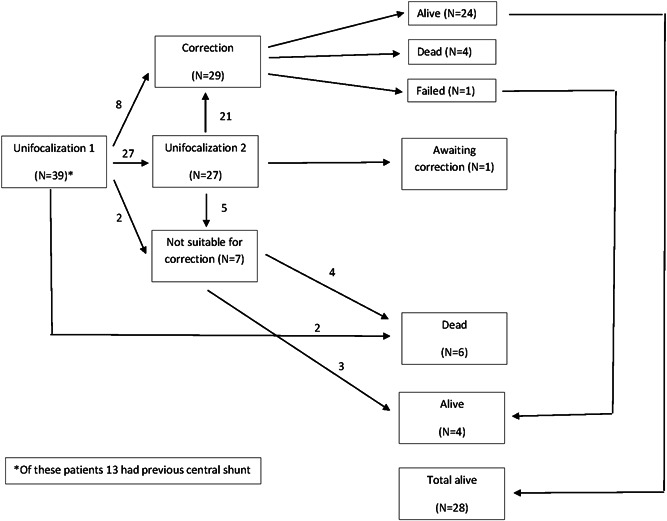
Flowchart of our patient population. Subsequent series of patients following the staged protocol of unifocalization and correction for pulmonary atresia, ventricular septal defect, and systemic‐pulmonary collateral arteries

Operative mortality was 3% (*n* = 1). This patient had a reoperation for leakage of the proximal suture of the homograft at the same day of the correction. However, due to extended cerebral hypoperfusion, the patient suffered a fatal cerebrovascular accident. Seven patients were not eligible for correction. In three patients this was due to pulmonary hypertension. One patient had malperfusion of the complete left lung, one patient had a hypoplastic right ventricle which was not suitable for repair, one patient had hypoplasia of the lungs and airways in combination a neuro‐cognitive disorder, and a further patient was considered to have an unacceptably high intraoperative risk of morbidity and mortality due to developmental disorders. Figure 1 shows a flowchart of our patient population.

In the 10 patients with 22q11 deletion, one is awaiting correction, three were ineligible (two died and one remains alive) for final correction, and six patients had a definitive correction. One patient died postoperatively due to a cerebrovascular accident. Compared to non‐syndromic patients, a lower percentage of the 22q11 deletion patients reached their final correction (23 out of 29 [79%]) versus 6 out of 9 (67%) respectively), although not statistically significant (*p* = .55).

### 3.4 Follow‐up

**Figure 2:**
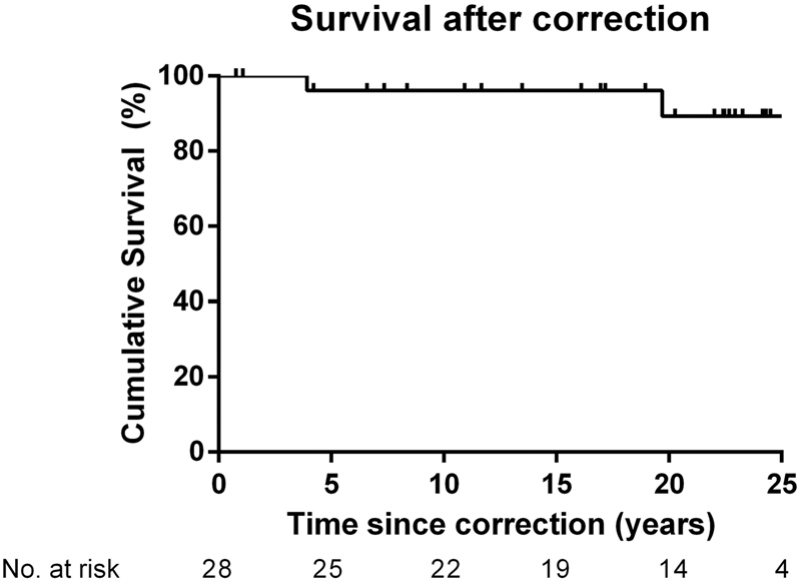
Kaplan–Meier curve of the survival after complete correction

Median follow‐up time after the correction was 19 years (range 1–27 years). Overall survival after definitive correction was 96% at 20 years (Figure 2). One patient died four years after successful correction of unknown cause, likely of a cardiac arrhythmia. Two patients died 20 years after correction due to progressive heart failure. Two of these patients had 22q11 deletion. From the survivors, all but one are in New York Heart Association Class I or II. Four of the seven patients who were not suitable for correction died. One patient died 2 years after the last unifocalization due to respiratory failure and infection. One patient died of unknown cause 9 years after the last unifocalization procedure. One patient died 14 years after the last unifocalization of multiorgan failure and sepsis and one patient died of massive intracranial bleeding 9 years after the last unifocalization.

Among the five survivors with 22q11 deletion, one is awaiting correction, one is palliated in a reasonable condition, three had a definitive correction (one in reasonable condition and two in good condition). The overall survival in the 22q11 deletion patient was significantly (*p* = .041) lower compared to nonsyndromic patients, (5 out of 10 [50%]) versus 25 out of 29 [67%], respectively).

#### 3.4.1 Interventions after correction

**Figure 3:**
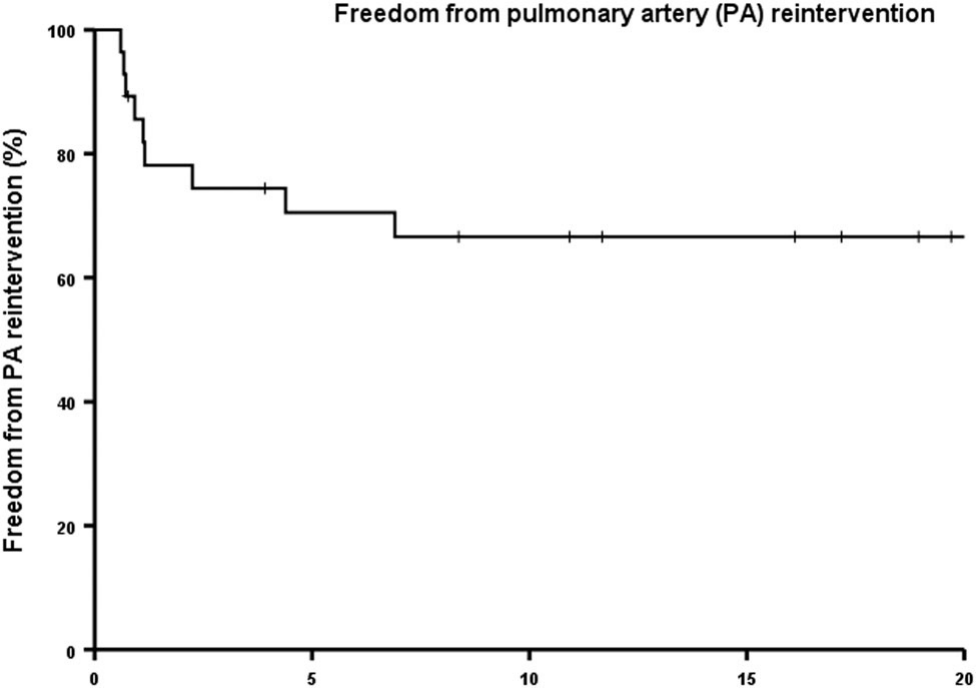
Kaplan–Meier curve of the freedom from reintervention on the pulmonary arteries

**Figure 4:**
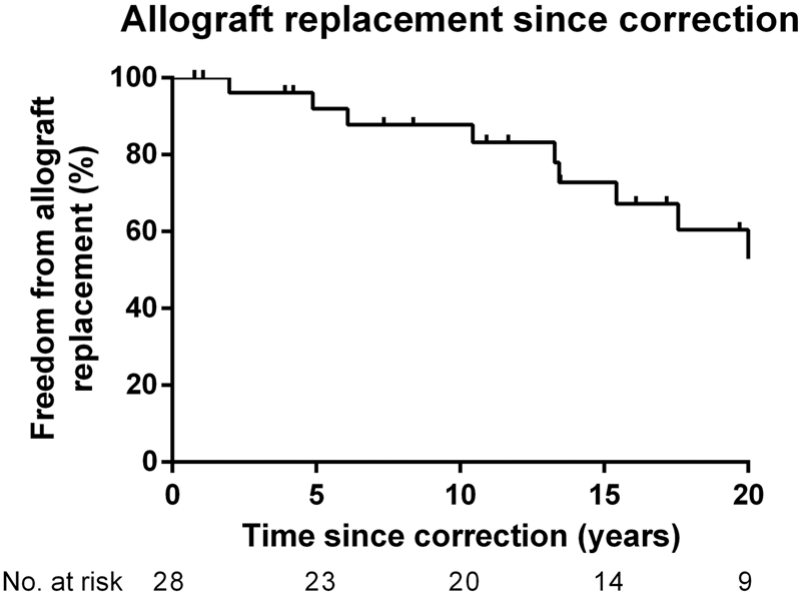
Kaplan–Meier curve of the freedom from pulmonary valve replacement after correction

After the final correction in 21 patients, other interventions were performed. They are listed in Table 1 and consist mostly of pulmonary valve replacement either surgically or percutaneously and dilatation or stenting of pulmonary branches. Freedom from interventions on the pulmonary arteries was 71% at 5 years and 67% at 10, 15, and 20 years (Figure 3). The modified Blalock Taussig shunt mentioned in Table 1 was placed in a patient with stenosis of a hypoplastic left pulmonary artery. Freedom from pulmonary valve replacement was 88%, 73%, 60%, and 27%% at 5, 10, 15, and 20 years, respectively (Figure 4).

**Table 1:** Surgical procedures and catheter interventions during follow‐up after successful correction

Patient	Intervention
1	Melody valve implantation
2	Closure residual VSD, patch augmentation AP, allograft replacement
3	Relief of RVOT obstruction
4	Balloon dilatation APS, allograft replacement, ascending aortic replacement, fenestrating ASD
5	MBT left, Melody valve implantation
6	Allograft replacement
7	Balloon dilatation and stent APD
8	Allograft replacement
9	Closure residual VSD, allograft replacement
10	Balloon dilatation APD, stenting APD and APS, Residual VSD, allograft implantation
11	Closure residual VSD, unifocalization right, Melody valve implantation
12	Allograft replacement
13	Stenting APS
14	Closure residual VSD
15	Balloon dilatation and stent APD
16	Resternotomy for bleeding, stent APD and APS, allograft replacement and augmentation APD
17	Allograft replacement
18	Stenting APS, balloon dilatation stent
19	Stenting APS and APD
20	Coil closure SPCA left

Abbreviations: AP, pulmonary artery; APD, right pulmonary artery; APS, left pulmonary artery; MBT, modified Blalock–Taussig shunt; RVOT, right ventricular outflow tract; SPCA, systemic‐to‐pulmonary collateral artery; VSD, ventricular septal defect.

#### 3.4.2 Echocardiography

Echocardiographic data after correction at the last check, showed in 18 patients (75%) a reasonable or good right ventricular function (RVF). Four patients (17%) had a moderate RVF. Only two patients (8%) had a severely impaired RVF.

The tricuspid regurgitation was trivial, mild, and moderate in 10 (42%), 10 (42%) and four patients (16%), respectively. The pulmonary regurgitation was absent/trivial, mild, moderate, or severe in 6 (26%), 8 (35%), 6 (26%), and 3 (13%), respectively. The right ventricular dilatation was absent, mild, moderate, and severe in 2 (8%), 4(17%), 12 (50%), and 6 (25%), respectively. If measurable, the median right ventricular (RV) pressure was estimated at median of 54 (25–108) mmHg. The median estimated pressure across the homograft is 19 (7–49) mmHg. Further to this, the calculated pressure differences of 32 (0–95) mmHg suggest increased pulmonary artery pressures.

In seven patients a small residual VSD was present, without hemodynamic significance in terms of flow.

#### 3.4.3 MR imaging

From 17 patients after correction (61%) we obtained detailed MR imaging with a median interval between correction and MR image of 15.6 years (range 9–22 years). Using these images, the estimated median right ventricular ejection fraction (RVEF) was calculated at 44% (range 13%–62%), the median left ventricular ejection fraction (LVEF) was 52% (range 29%–64%), the median RV end‐diastolic volume was 190 ml (range 94–339 ml), indexed 105 ml/m^2^ (range 76–176 ml/m^2^) and the median pulmonary regurgitation fraction was 19% (range 0%–50%).

## 4 DISCUSSION

In patients with PA, VSD, and SPCAs, several different strategies have been reported over the last 30 years, indicating the challenging management of these conditions.^2^, ^13^, ^14^, ^15^, ^16^ In 1989 we selected a single strategy for all subsequent patients, and have maintained the use of this protocol ever since. This allows to present our long‐term results after 30 years, demonstrating that 81% of the patients surviving after unifocalization are suitable for biventricular correction with a conduit between RVOT and the unfocalized pulmonary artery system. We consider this an adequate success rate in the complete and unselected cohort of these complex patients, also compared to other series and strategies.^2^, ^14^, ^17^, ^18^, ^19^, ^20^, ^21^ The use of a central aorto‐pulmonary shunt to promote growth in diminutive pulmonary arteries is still the subject of several studies.^15^, ^22^ In a preliminary report of our first six cases, we concluded that central shunts did not preclude the need for unifocalization with no significant growth of the pulmonary arteries.^23^

Over the years of working with the staged approach, several aspects have evolved. We reduced the diameter of the MBT shunts as a result of our experience with this procedure. We learned to avoid as much tissue of the SPCAs as possible. Closure of unnecessary collateral arteries and intrapulmonary anastomoses are two factors that prevent future issues with stenosis or dilatation. Nevertheless, these problems do occur due to the histological characteristics of the collateral arteries.^10^ During follow‐up several dilatation and stenting procedures may be necessary and, in some cases, adequate relieve of stenosis cannot be maintained in the longer term.

As a result of our initial experience with central shunts with disappointing results in terms of growth and increase of the pulmonary artery size,^23^ we use this procedure for diminutive pulmonary arteries to promote outgrowth as mentioned by other authors.^24^, ^25^ In our opinion, the rehabilitation of the pulmonary artery as promoted by several authors^15^, ^26^, ^27^ may be useful, but still does not preclude further unifocalization in most of our cases.

Our current approach consists of early CT scanning after echocardiographic diagnosis. In the case of diminutive central pulmonary arteries, a central shunt is constructed. Evaluation is performed at the end of the first year of life. This evaluation, after central shunting performed in the case of small to normal confluent pulmonary arteries, a system of diagnostic cardiac catheterization (HC) is employed to document the pulmonary arterial system and all SPCAs including their relation to the native pulmonary arteries.

Depending on the individual anatomy, unilateral unifocalization is performed through lateral thoracotomies with the occlusion of SPCAs providing dual supply, and with the incorporation of SPCAs into a single supply. Incorporation preferably is done at the level of the interlobar arteries, to exclude as much extra‐pulmonary SPCA vessel tissue as possible. During the first unifocalization, an additional MBT shunt is commonly constructed. After repeated cardiac catherization, a second contralateral unifocalization is performed if necessary shortly thereafter, with an MBT shunt only constructed if indicated. Between 1.5 and 2 years of age and with acceptable pulmonary arterial pressures and anatomy, the final correction is completed, often with augmentation of the confluent pulmonary arteries.

During long‐term follow‐up allograft degeneration and replacement occurred as expected. An explanation for this could be that these allografts are placed in a more extra‐anatomical position, in patients with a younger mean age at correction. Also elevated right ventricular pressures, probably due to an abnormal pulmonary vascular bed, can contribute to this degenerative process.^28^

Considering the complexities of this patient group, we are cognizant that these patients’ life expectancies fall below normal, but lack long‐term data comparable with others with right‐sided allograft placement, that is, tetralogy of Fallot.^29^

This study is limited by the relatively small group of patients. However, we treated all cases consecutively and without selection over the course of 30 years, employing an identical strategy by the same team. Given the limited case numbers, we did not perform sub‐analysis for example for patients operated on at the beginning of the case series, versus those who underwent this process more recently, after the team had gained considerable experience in the interim.

In conclusion, staged repair of PA, VSD, and SPCAs offers an adequate solution for most patients with a high correction rate and low operative mortality. Surgical or catheter‐based interventions are necessary during follow‐up for pulmonary artery stenosis and allograft degeneration. Long‐term survival appears to be diminished.

We continue to follow these patients to collect more data on long‐term outcomes. The function of the right ventricle is key a point of concern, as echocardiography and cardiac MRI show moderate and severe dilatation of the right ventricle (75%) and impairment of the function in 25% of the patients. Additional genetic abnormalities, in particular 22q11 deletion, appears to be risk factor for adverse outcomes.^30^

Another limitation is the retrospective collection of data which results in incomplete data on exercise testing and quality of life studies. We are also looking to prospectively compare our long‐term results to those of other strategies, to facilitate a better comparison between the different methods for the management of this challenging patient group.
